# Genotypic and phenotypic evaluation of off-type grasses in hybrid Bermudagrass [*Cynodon dactylon* (L.) Pers. x *C. transvaalensis* Burtt-Davy] putting greens using genotyping-by-sequencing and morphological characterization

**DOI:** 10.1186/s41065-017-0043-3

**Published:** 2017-08-18

**Authors:** Eric H. Reasor, James T. Brosnan, Margaret E. Staton, Thomas Lane, Robert N. Trigiano, Phillip A. Wadl, Joann A. Conner, Brian M. Schwartz

**Affiliations:** 1 Department of Plant and Soil Sciences, Mississippi State University, 117 Dorman Hall, 32 Creelman Street, Mississippi State University, Mississippi State, MS 39762 USA; 2 Department of Plant Sciences, University of Tennessee, 2431 Joe Johnson Dr., 252 Ellington Plant Sciences Bldg., Knoxville, TN 37996 USA; 3 Department of Entomology and Plant Pathology, University of Tennessee, 2505 E.J. Chapman Dr., 370 Plant Biotechnology Bldg, Knoxville, TN 37996 USA; 4 United States Department of Agriculture, Agriculture Research Service, United States Vegetable Laboratory, 2700 Savannah Highway, Charleston, SC 29414 USA; 5 Department of Horticulture, University of Georgia, 2360 Rainwater Rd, Tifton, GA 31794 USA; 6 Department of Crop and Soil Sciences, University of Georgia, University of Georgia, 2360 Rainwater Rd, Tifton, GA 31794 USA

**Keywords:** Turfgrass, Bermudagrass, Off-types, Genotyping-by-sequencing, Morphology, Putting greens

## Abstract

**Background:**

Interspecific hybrid bermudagrass [*Cynodon dactylon* (L.) Pers. x *C. transvaalensis* Burtt-Davy] is one of the most widely used grasses on golf courses, with cultivars derived from ‘Tifgreen’ or ‘Tifdwarf’ particularly used for putting greens. Many bermudagrass cultivars established for putting greens can be genetically unstable and lead to the occurrence of undesirable off-type grasses that vary in phenotype. The objective of this research was to genetically and phenotypically differentiate off-type grasses and hybrid cultivars. Beginning in 2013, off-type and desirable hybrid bermudagrass samples were collected from golf course putting greens in the southeastern United States and genetically and phenotypically characterized using genotyping-by-sequencing and morphology.

**Results:**

Genotyping-by-sequencing determined that 11% (5) of off-type and desirable samples from putting greens were genetically divergent from standard cultivars such as Champion, MiniVerde, Tifdwarf, TifEagle, and Tifgreen. In addition, genotyping-by-sequencing was unable to genetically distinguish all standard cultivars from one another due to their similar origin and clonal propagation; however, over 90,000 potentially informative nucleotide variants were identified among the triploid hybrid cultivars.

**Conclusions:**

Although few genetic differences were found in this research, samples harvested from golf course putting greens had variable morphology and were clustered into three distinct phenotypic groups. The majority of off-type grasses in hybrid bermudagrass putting greens were genetically similar with variable morphological traits. Off-type grasses within golf course putting greens have the potential to compromise putting surface functionality and aesthetics.

## Background

The economic impact of the golf industry in the United States (U.S.) in 2011 was estimated to be $176.8 billion with a contribution of approximately 1.98 million jobs (SRI International; http://wearegolf.org/economy/impact). Interspecific hybrid bermudagrasses (*Cynodon dactylon* (L.) Pers. x *C. transvaalensis* Burtt-Davy) are some of the most widely utilized grasses on golf courses throughout tropical, subtropical, and temperate climates due to their desirable turfgrass characteristics of texture, color, and stress tolerance [2]. Putting greens are a vital aspect of a golf course and in 2007 hybrid bermudagrasses were grown on 80% of putting green acreage in the southern U.S. [29].

‘Tifgreen’ was one of the first interspecific hybrids developed for putting green use [6, 21]. Soon after its commercial release, ‘Tifdwarf’ was selected from a somatic mutation in a ‘Tifgreen’ establishment [7, 8]. Despite being genetically unstable [9, 10], ‘Tifgreen’ and ‘Tifdwarf’ are used on putting surfaces; however, superior mutations have been released as new “ultradwarf” cultivars [36]. Ultradwarf bermudagrass cultivars are classified due to their more diminutive morphology (i.e., internode length and leaf length and width) compared to ‘Tifdwarf’. ‘Champion’ and ‘MiniVerde’ were selected from somatic mutations in established ‘Tifdwarf’ plantings [5, 23], whereas ‘TifEagle’ was a putative mutant from radiation-induced ‘Tifgreen’ or ‘Tifway II’ rhizome [18, 20, 41]. In all cases, “ultradwarf” cultivars such as ‘Champion’, ‘MiniVerde’, and ‘TifEagle’ were once identified as off-types.

Morphological characterization has been considered a traditional method of studying turfgrass classification and diversity [3, 22, 25, 38]. Morphological characteristics such as internode length, leaf length, leaf width, and stolon diameter are of particular interest for classification of bermudagrasses [25, 26, 38] because differences in morphology can differentiate off-types from desirable cultivars [9, 10]. Additionally, Roche and Loch [37] stated that morphological characterization could provide useful information to further research for adaptation and management of different hybrid bermudagrasses. Researchers have used morphological characterizations to compare hybrid bermudagrass cultivars within the ‘Tifgreen’ family [31, 37]; however, morphological inconsistencies suggest that molecular techniques are also needed to accurately evaluate hybrid bermudagrass diversity [36].

**Fig. 1:**
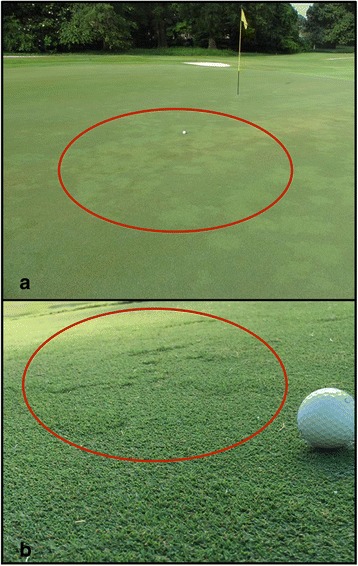
**a** Off-type grasses (lighter in color and noted by red circle) present in an ultradwarf bermudagrass (*Cynodon dactylon* (L.) Pers. x *C. transvaalensis* Burtt-Davy) putting green. The difference in turfgrass color between desirable and off-type grasses disrupts aesthetic uniformity of the putting surface. **b** Close-up of an off-type grass patch (noted by red circle) present in an ultradwarf bermudagrass putting green. The difference in growth rate between the desirable and off-type grasses has the potential to disrupt the functional uniformity of putting surfaces with off-type infestations. Figure was generated using Keynote (v6.6.2)

Several methods have been used to explore genotypic differences among off-type grasses and hybrid bermudagrass cultivars. DNA amplification fingerprinting (DAF) and signatures from amplification profiles identified contaminant off-types not related to the ‘Tifgreen’ family, but could not distinguish mutant off-types within the ‘Tifgreen’ family [9]. Amplified fragment length polymorphisms (AFLPs) studies determined the genetic diversity among several ‘Tifgreen’-derived cultivars. Studies utilizing AFLPs grouped ‘Tifgreen’, ‘Tifdwarf’, ‘TifEagle’, and ‘Champion’ into the same genetic cluster despite the grasses having differing phenotypic characteristics [11, 12, 41]. Attempts to use various simple-sequence repeats (SSRs) to identify ‘Tifgreen’-derived hybrid bermudagrass cultivars met with limited success as well [19, 24]. While SSRs identified ‘TifEagle’ from other ‘Tifgreen’-derived cultivars and identified polymorphisms unique to ‘Tifdwarf’ and ‘MiniVerde’ [19, 24], SSRs are not able to readily distinguish all ‘Tifgreen’-derived hybrid bermudagrass cultivars from one another nor have they been able to identify weedy off-type grasses from standard cultivars used on golf course putting greens. Fig. 1 displays the aesthetic and functional disruptions that off-type grasses in bermudagrass putting greens can create.

Genotyping-by-sequencing (GBS) is a high-throughput, next generation sequencing method capable of generating large numbers of single nucleotide polymorphism (SNPs) from species with high diversity [13]. GBS offers several advantages over other molecular marker techniques including the amount data generated and the price per sample [13, 15]. Moreover, GBS allows analysis of a species (e.g., hybrid bermudagrass) for which a complete reference genome sequence is not available. Fiedler et al. [15] identified over 4600 high-quality SNPs in switchgrass (*Panicum virgatum*) using an early draft genome assembly with only half of the assembled DNA contigs scaffolded. Poland et al. [32] used GBS to map over 34,000 SNPs for the Oregon Wolfe Barley (*Hordeum vulgare*) reference population and 20,000 SNPs for the Synthetic W9784xOpata85 wheat (*Triticum aestivum*) reference population, both of which lacked a complete reference genome sequence. Based on the robustness of the technique and successful use in other grasses without a complete reference genome [13, 15, 32, 33], we hypothesize that GBS may be able to identify genetic variation among off-types and hybrid bermudagrasses used on putting greens. Therefore, our objectives were to explore the genetic and the phenotypic variation among off-type grasses sampled from hybrid bermudagrass putting greens using GBS and morphological characterization.

## Methods

Single replicates of desirable and off-type hybrid bermudagrass samples were harvested in 2013 from putting greens on golf courses in Alabama, Arkansas, Florida, Mississippi, South Carolina, and Tennessee (Table 1). The greenkeeper at each golf course determined samples that were desirable from those that were off-type grasses. Samples were harvested with a 7.5 cm diameter tubular plugger (Turf Tec International; Tallahassee, FL, USA) and established using one three node stolon planted in a 64 cm^2^ pot filled with a peat moss based growing medium (Pro-Mix BX Mycorrhizae; Premier Horticulture, Inc.; Quakertown, PA, USA) in a glasshouse environment at the University of Tennessee (Knoxville, TN, USA; 35.5°N, −83.5°W). Plants were maintained with 24 kg N ha^−1^ wk.^−1^ of a water-soluble complete fertilizer (20 N-8.7P-16.6 K; Southern Agriculture; Hendersonville, NC, USA), irrigated to promote active growth, and insecticides (abamectin 0.01 kg ai ha^−1^, Avid 0.15EC, Syngenta; pymetrozine 0.35 kg ai ha^−1^, Endeavor, Syngenta) were applied on a preventive basis.

**Table 1:** Plant material used in genetic and phenotypic evaluation of off-type grasses in ultradwarf hybrid bermudagrass putting greens (*Cynodon dactylon* (L.) Pers. x *C. transvaalensis* Burtt-Davy)

Sample	Sample Origin^a^	USA State	Ploidy^b^
S1	Champion (DS)	TN	2n = 3× = 27
S2	Champion (DS)	MS	2n = 3× = 27
S3	Champion (DS)	TN	2n = 3× = 27
S4	Champion (DS)	TN	2n = 3× = 27
S5	Champion (DS)	TN	2n = 3× = 27
S6	Champion (DS)	TN	2n = 3× = 27
S7	Champion (DS)	TN	2n = 3× = 27
S8	Champion (DS)	MS	2n = 3× = 27
S9	Champion (DS)	TN	2n = 3× = 27
S10	Champion (DS)	MS	2n = 3× = 27
S11	Champion (DS)	AR	2n = 3× = 27
S12	Champion (DS)	TN	2n = 3× = 27
S13	MiniVerde (DS)	FL	2n = 3× = 27
S14	MiniVerde (DS)	FL	2n = 3× = 27
S15	MiniVerde (DS)	TN	2n = 3× = 27
S16	MiniVerde (DS)	TN	2n = 3× = 27
S17	TifEagle (DS)	AL	2n = 3× = 27
S18	TifEagle (DS)	TN	2n = 3× = 27
S19	MiniVerde (OT)	FL	2n = 3× = 27
S20	MiniVerde (OT)	FL	2n = 3× = 27
S21	Champion (OT)	TN	2n = 3× = 27
S22	Champion (OT)	MS	2n = 3× = 27
S23	Champion (OT)	TN	2n = 3× = 27
S24	Champion (OT)	TN	2n = 3× = 27
S25	Champion (OT)	TN	2n = 3× = 27
S26	Champion (OT)	TN	2n = 3× = 27
S27	Champion (OT)	TN	2n = 3× = 27
S28	Champion (OT)	TN	2n = 3× = 27
S29	Champion (OT)	TN	2n = 3× = 27
S30	Champion (OT)	TN	2n = 3× = 27
S31	Champion (OT)	MS	2n = 3× = 27
S32	Champion (OT)	MS	2n = 3× = 27
S33	Champion (OT)	TN	2n = 3× = 27
S34	Champion (OT)	MS	2n = 3× = 27
S35	Champion (OT)	AR	2n = 3× = 27
S36	Champion (OT)	TN	2n = 3× = 27
S37	Champion (OT)	TN	2n = 3× = 27
S38	MiniVerde (OT)	TN	2n = 3× = 27
S39	TifEagle (OT)	AL	2n = 3× = 27
S40	TifEagle (OT)	AL	2n = 3× = 27
S41	MiniVerde (OT)	TN	2n = 3× = 27
S42	MiniVerde (DS)	TN	2n = 3× = 27
S43	MiniVerde (OT)	FL	2n = 3× = 27
S44	MiniVerde (OT)	MS	2n = 3× = 27
S45	MiniVerde (OT)	MS	2n = 3× = 27
S46	MiniVerde (OT)	TN	2n = 3× = 27
S47	TifEagle (DS)	AL	2n = 3× = 27
S48	TifEagle (DS)	AL	NA
S49	Champion (OT)	TN	NA
S50	Champion (OT)	TN	NA
S51	Champion (OT)	GA	NA
S52	Champion (OT)	GA	NA
S53	Champion (OT)	TN	NA
S54	Champion (OT)	TN	NA
S55	MiniVerde (DS)	TN	NA
S56	Champion (DS)	SC	NA
S57	Champion (DS)	GA	NA
S58	TifEagle (OT)	MS	NA
S59	Champion (OT)	GA	NA
S60	TifEagle (DS)	TN	NA
S61	TifEagle (DS)	MS	NA
S62	MiniVerde (DS)	TN	NA
CH1–6	Champion (ST)	GA	2n = 3× = 27
MV1–6	MiniVerde (ST)	GA	2n = 3× = 27
TD1–6	Tifdwarf (ST)	GA	2n = 3× = 27
TE1–6	TifEagle (ST)	GA	2n = 3× = 27
TG1–6	Tifgreen (ST)	GA	2n = 3× = 27
TW1–6	Tifway (ST)	GA	2n = 3× = 27
TA1–3	*C. dactylon* (ST)	GA	2n = 4× = 36
TB1–3	*C. dactylon* (ST)	GA	2n = 4× = 36
DA1–3	*C. transvaalensis* (ST)	GA	2n = 2× = 18
DB1–3	*C. transvaalensis* (ST)	GA	2n = 2× = 18

Selections included 62 desirable (DS) and off-type (OT) ultradwarf bermudagrasses sampled from golf course putting greens in TN, MS, AR, FL, AL, GA, and SC. Six standard (ST) hybrid bermudagrass cultivars [Champion (CH1–6), MiniVerde (MV1–6), Tifdwarf (TD1–6), TifEagle (TE1–6), Tifgreen (TG1–6), and Tifway (TW1–6)] and two progenitor species [(*C. dactylon* (TA1–3 and TB1–3) and *C. transvaalensis* (DA1–3 and DB1–3)] were included in the analysis for comparison. Ploidy level was confirmed using flow cytometry

^a^Desirable and off-type samples were harvested from golf course putting greens. Standard samples were provided by the University of Georgia Coastal Plain Experiment Station in Tifton, GA

^b^Ploidy was confirmed using flow cytometry. Ploidy was not confirmed for samples with “NA”

Ploidy levels were confirmed for each sample included in the GBS assay using flow cytometry at the University of Georgia Coastal Plain Experiment Station (Tifton, GA). Fresh leaf tissue was isolated from samples and chopped using a razor in 300 μL of LB01-lysis buffer (15 mM Tris, 2 mM Na_2_EDTA, 0.5 mM spermine-4HCl, 80 mM KCl, 20 mM NaCl, 0.1% *v*/v Triton X-100 pH 7.5 and 16 mM ß-mercaptoethanol) to release nuclei. Each bermudagrass sample was combined with *Sorghum bicolor* cv. BTx623 for a standard genome size comparison. Samples were passed through a 30-μm filter (CellTrics; Partec; Munster, Germany) and then 150 μL of RNase and propidium iodide solution (PI/RNase Staining Buffer, BD Biosciences, San Jose, CA, USA) was added. Samples were incubated on ice for 15 min and analyzed on an Accuri C6 flow cytometer (BD Biosciences; San Jose, CA, USA). Gating was set by the selection of objects that exhibited a strong correlation between the FL2 and FL3 signals using a flow rate of 14 μL per minute and a minimum cell count of 10,000. The mean FL2-A peaks from the signals were determined for *S. bicolar* and each unknown hybrid bermudagrass sample using Accuri C6 software (BD Biosciences; San Jose, CA, USA). These mean FL2-A values were then used with *S. bicolar* genome size (1.67 pg/2C) to calculate the genome size of each unknown hybrid bermudagrass sample [34].

### Genotyping-by-sequencing

#### Plant material and DNA isolation

Desirable and off-type samples labeled S1 to S47 in Table 1 were included in GBS due to the expense of the analysis. Hybrid bermudagrass cultivars [Champion (CH1–6), MiniVerde (MV1–6), Tifdwarf (TD1–6), TifEagle (TE1–6), Tifgreen (TG1–6), and Tifway (TW1–6)] and progenitor species [*C. dactylon* (TA1–3 and TB1–3) and *C. transvaalensis* (DA1–3 and DB1–3)] were used as standards in the GBS analysis. Only three biological replicates of the progenitor species were included also due to the expense of the analysis. Plant material for these standard entries was obtained from the University of Georgia Coastal Plain Experiment Station (Tifton, GA, USA).

For all samples, plant genomic DNA was isolated from actively growing leaf tissue on a single stolon using the Qiagen DNeasy Plant Mini Kit (Qiagen; Valencia, California, USA) according to the manufacturer’s protocol. DNA concentration was quantified using an intercalating dye (Quant-iT™ PicoGreen dsDNA Asasy Kit; Life Technologies; Carlsbad, CA, USA). DNA working solutions for the GBS protocol had a total volume of 30 μL and a concentration ranging from 50 to 105 ng μL^−1^.

#### Genotyping-by-sequencing analysis

Genotyping-by-sequencing was conducted at the Cornell University Institute for Biotechnology (Ithaca, NY) using the protocol described by Elshire et al. [13]. *Ape*KI restriction enzyme was selected based on optimization trials for the GBS digestion to maximize the number of sampled genomic loci [13]. Libraries for next-generation sequencing were constructed from DNA samples and multiplexed using Illumina HiSeq 2500 and then Illumina NextSeq 500 to increase read coverage and depth.

#### Bioinformatics analysis

The combined Illumina data sets were initially analyzed with the UNEAK pipeline of the Tassel software package [4, 17]. One of the limitations of the UNEAK pipeline is that its nucleotide variant calling algorithm relies on a diploid model. Bermudagrasses sequenced in our GBS analysis included diploid (2n = 2× = 18), triploid (2n = 3× = 27), and tetraploid (2n = 4× = 36) samples; therefore, an alternative approach was used to call variants. Sequence tags with a predicted variant were extracted from the topm.bin libraries generated during the Tag-Pair-Export phase using the UNEAK Binary-to-text-plugin; then raw reads were mapped with Bowtie2 v2.2.7 to these tags generated from the UNEAK pipeline as a pseudo-reference [27].

The haplotype-based variants caller, Freebayes v1.0.2–15, was used to call variants for each set of samples with the same ploidy level with the correct ploidy level specified with parameter p [16]. The sorting, indexing, and merging of alignment files was performed with the SAMtools v1.3 package [28]. Multidimensional scaling (MDS) plots were generated from these variants using PLINK v1.9 to illustrate the variation among samples. The bermudagrass samples S19, S28, S30, S32, and S44 were not included because they had less than one million raw-reads [35]. The individual samples for each triploid cultivar were pooled to increase the read depth for each cultivar. The read alignment files were pooled using SAMtools v1.3 package [28] and then the FreeBayes method of determining variants was utilized again. The pooled data was then used in a custom Python script to determine loci that differed between at least two cultivars (github.com/statonlab/UDBG_Informative_SNPs/blob/master/find_informative_SNPs.py). Any loci with three different genotypes (homozygous for the reference allele, homozygous for the alternate allele, or heterozygous) for at least two cultivars were flagged. Loci were not flagged if heterozygosity differed between or within the two subgenomes in triploid cultivars (i.e., 0/0/1 or 0/1/1). Lastly, each cultivar was filtered to use only individual variants with a read depth greater than or equal to 40. All raw read data has been submitted to NCBI under BioProject accession PRJNA353769.

### Phenotypic evaluation

#### Plant materials

Bermudagrass samples labeled S1 to S62, except for S13, S14, S19, S20, and S43 were used in phenotypic evaluation (Table 1). Bermudagrass samples S13, S14, S19, S20, and S43 were excluded from phenotypic evaluation due to their harvest date. The greenkeeper at each golf course determined samples that were desirable from those that were off-type grasses. Samples were harvested with a 7.5 cm diameter tubular plugger (Turf Tec International; Tallahassee, FL, USA) and established during the summer of 2013 using one three node stolon planted in a 64 cm^2^ pot filled with a peat moss based growing medium (Pro-Mix BX Mycorrhizae; Premier Horticulture, Inc.; Quakertown, PA, USA) in a glasshouse environment at the University of Tennessee (Knoxville, TN, USA; 35.5°N, −83.5°W). Plants were maintained with 24 kg N ha^−1^ wk.^−1^ of a water-soluble complete fertilizer (20 N-8.7P-16.6 K; Southern Agriculture; Hendersonville, NC, USA), irrigated to promote active growth, and insecticides (abamectin 0.01 kg ai ha^−1^, Avid 0.15EC, Syngenta; pymetrozine 0.35 kg ai ha^−1^, Endeavor, Syngenta) were applied on a preventive basis.

For phenotypic evaluation, one three-node stolon of each sample was established in four, 64 cm^2^ pots filled with a peat moss based growing medium (Pro-Mix BX Mycorrhizae; Premier Horticulture, Inc.; Quakertown, PA, USA) on 7 April 2014. The stolon length at transplanting of the 52 selections ranged from 3.4 to 11.3 cm. The plants were maintained as previously described but regular clipping was ceased 2 weeks prior to evaluation.

#### Morphological measurements and statistical analysis

Phenotypic evaluation of off-type and desirable samples was conducted by measuring plant morphological characteristics via methods outlined by Roche and Loch [37]. Five parameters were assessed and included internode length and stolon diameter, leaf length and width, and the leaf length:width ratio (LWR). Measurements were made between the third and fourth node and on the outer leaf from the third node using digital calipers (Digimatic Caliper, Model No.CD-6″ CX, Mitutoyo Corporation, Kawasaki-shi, Kanagawa, Japan). The experiment was a completely randomized design with pots replicated four times and morphology measured on three stolons per pot. Morphology was assessed on 3 June 2014 and repeated again on 25 June 2014.

All morphological data describing desirable and off-type hybrid bermudagrass samples were analyzed using cluster analysis in SAS Enterprise Guide (Version 6.1, SAS Institute, Cary, NC, USA). K-means clustering algorithm was used to partition the data set into a user-defined number of clusters [30]. Three clusters were determined based on the cubic clustering criterion and the frequency of observations in each cluster [30]. Cluster means and standard deviations for each morphological measurement were then graphed in Prism (Prism 6 for Mac OS X; GraphPad Software, Inc.) to determine statistical differences among cluster means.

## Results

### Genotyping-by-sequencing

**Fig. 2:**
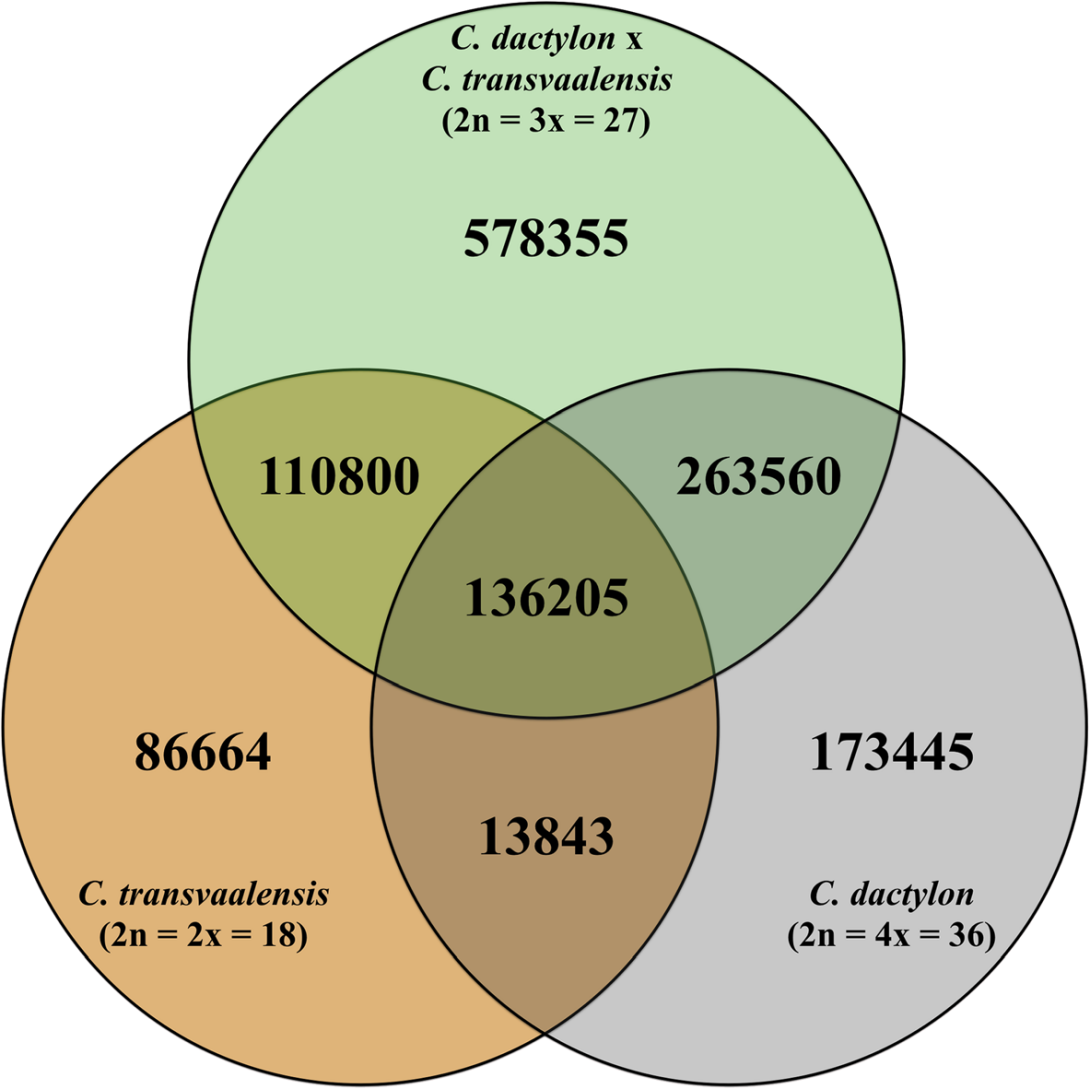
Venn diagram showing the number of total and shared nucleotide variants from GBS for *Cynodon transvaalensis*, *C. dactylon*, and *C. dactylon* x *C. transvaalensis.* Ploidy levels were confirmed using flow cytometry and are noted in parentheses. Figure was generated using Keynote (v.6.6.2)

Over 878 million reads were generated through Illumina sequencing, with 271 million from the Illumina HiSeq platform and an additional 606 million from the same libraries run on the Illumina NextSeq platform. Of the 878 million reads, 757 million were determined to be of high quality and were able to be demultiplexed and assigned to individual plant samples. After excluding five samples with less than a million reads, the remaining individual samples had a range of 1.3 (sample S44) million reads to 13.9 million reads (sample S22). The variants yielded from GBS analysis include single nucleotide variants, multiple nucleotide variants, and indels. An initial 1,088,920 total variants were identified with an average read depth of 4.9 sequences for the triploid bermudagrass (*C. dactylon* x *C. transvaalensis*) samples. GBS analysis identified 347,512 total variants with an average read depth of 9.5 per individual for the two diploid, *C. transvaalensis* selections. For the tetraploid, *C. dactylon* samples, 587,053 total variants were identified with an average sequence read depth of 7.4 (Fig. 2). Only 136,205 variants were shared among diploid, triploid, and tetraploid species; therefore, the remaining variants are fixed in at least one species (Fig. 2).

**Fig. 3:**
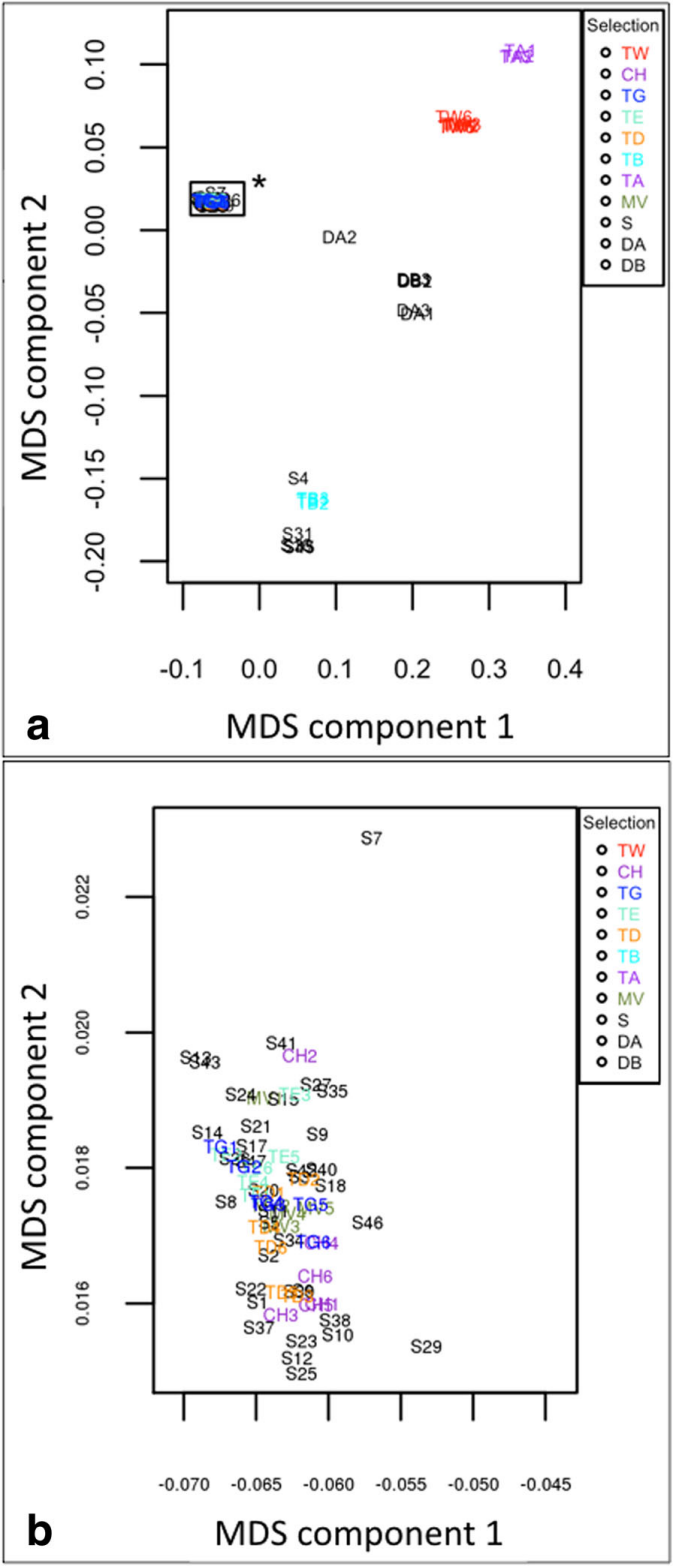
**a** Multidimensional scaling plot (MDS) of nucleotide variants from 47 desirable and off-type bermudagrasses sampled from golf course putting greens (S1–47), six hybrid bermudagrass (*Cynodon dactylon* (L.) Pers. x *C. transvaalensis* Burtt-Davy) cultivars [Champion (CH1–6), MiniVerde (MV1–6), Tifdwarf (TD1–6), TifEagle (TE1–6), Tifgreen (TG1–6), and Tifway (TW1–6)], and two progenitor species [*C. dactylon* (TA1–3, TB1–3) and *C. transvaalensis* (DA1–3, DB1–3)]. Samples S19, S28, S30, S32, and S44 were not included due to lack of read depth. Variants were generated using Freebayes, the MDS plot was calculated in Plink, and plotted in R. The asterisk on the box indicates the zoomed region in (**b**). **b** A blown up view of the boxed in region of (**a**) indicated by an asterisk. Desirable and off-type bermudagrasses that were analyzed by GBS but did not cluster in this region included the following samples: S4, S16, S31, S33, and S45

The majority of samples harvested from golf courses clustered with the hybrid bermudagrass cultivars Champion, MiniVerde, Tifdwarf, TifEagle, and Tifgreen in the MDS plot (Fig. 3). The clustering suggested that samples from golf courses were genetically similar to those hybrid bermudagrass cultivars typically established on golf course putting greens (Fig. 3). Of the 47 unknown samples, only five (~11%) were genetically divergent from the standard cultivars (S4, S16, S31, S33, and S45), as illustrated by the MDS plot (Fig. 3). Hybrid bermudagrass cultivars Champion, MiniVerde, Tifdwarf, TifEagle, and Tifgreen were genetically similar to one another; however, GBS separated these cultivars from ‘Tifway’ hybrid bermudagrass (Fig. 3). Pooling individual cultivar samples yielded a higher average read depth of 31 per variant site per cultivar. Using the pooled data, 675,578 loci were identified as different between at least two cultivars. The majority of these genotype differences were only able to differentiate cultivars within the ‘Tifgreen’-cultivar family from those with different lineage (i.e., ‘Tifway’) (Table 2). Table 2 exhibits the number of nucleotide variants with different genotypes between each pair of triploid hybrid bermudagrass cultivars. Variants were included if they were homozygous for the reference allele in one cultivar and homozygous for the alternate allele in the other cultivar or if they were homozygous in one cultivar and heterozygous in the other. ‘Tifway’ has a much larger number of variants compared to the other five cultivars (29,614 variants when compared to ‘MiniVerde’ to 37,802 when compared to ‘Tifdwarf’). The five other cultivars are more similar to each other, with the highest number of identified variants of 4476 between ‘Tifdwarf’ and ‘Tifgreen’.

**Table 2:** The number of nucleotide variants with different genotypes between each pair of triploid hybrid bermudagrass (*Cynodon dactylon* x *C. transvaalensis*) cultivars

Cultivar	Number of nucleotide variants				
	Champion	MiniVerde	Tifdwarf	TifEagle	Tifgreen
Champion	-	-	-	-	-
MiniVerde	4003	-	-	-	-
Tifdwarf	4086	3404	-	-	-
TifEagle	4281	3489	4299	-	-
Tifgreen	3969	3028	4476	4088	-
Tifway	35,104	29,614	37,802	36,796	36,838

The variants were filtered to loci with a read depth of at least 40 but less than 100 per cultivar. Variants were included if they were homozygous for the reference allele in one cultivar and homozygous for the alternate allele in the other cultivar or if they were homozygous in one cultivar and heterozygous in the other

The pooled data from variants among triploid cultivars still encompassed individual loci with both very low and very high individual read depths. Low read depth could miss heterozygotes, whereas high read depth could indicate a repetitive region instead of an individual locus. To mitigate read depth issues, a further filter was applied to identify only the most robust variants with a sequence read depth of at least 40, but no more than 100. The upper limit was set to filter reads originating from repetitive elements where detected variation is not likely to be from a single locus. Filtering using these read depths yielded 93,188 variants between at least two genotypes (Table 2).

The MDS plot revealed clear clustering of the diploid (DA1–3 and DB1–3) and tetraploid progenitor species (TA1–3 and TB1–3) apart from the standard hybrid bermudagrass cultivars (CH1–6, MV1–6, TD1–6, TE1–6, TG1–6, and TW1–6) and the majority of samples harvested from putting greens (Fig. 3a). The two progenitor species also clustered separately from one another with the exception of DA2 due to possible contamination during DNA isolation. The clustering demonstrated that GBS was effective for distinguishing diploid, triploid, and tetraploid bermudagrasses. The ability to distinguish among bermudagrass species is likely due to the large number of unshared variants (Fig. 2).

### Phenotypic evaluation

**Fig. 4:**
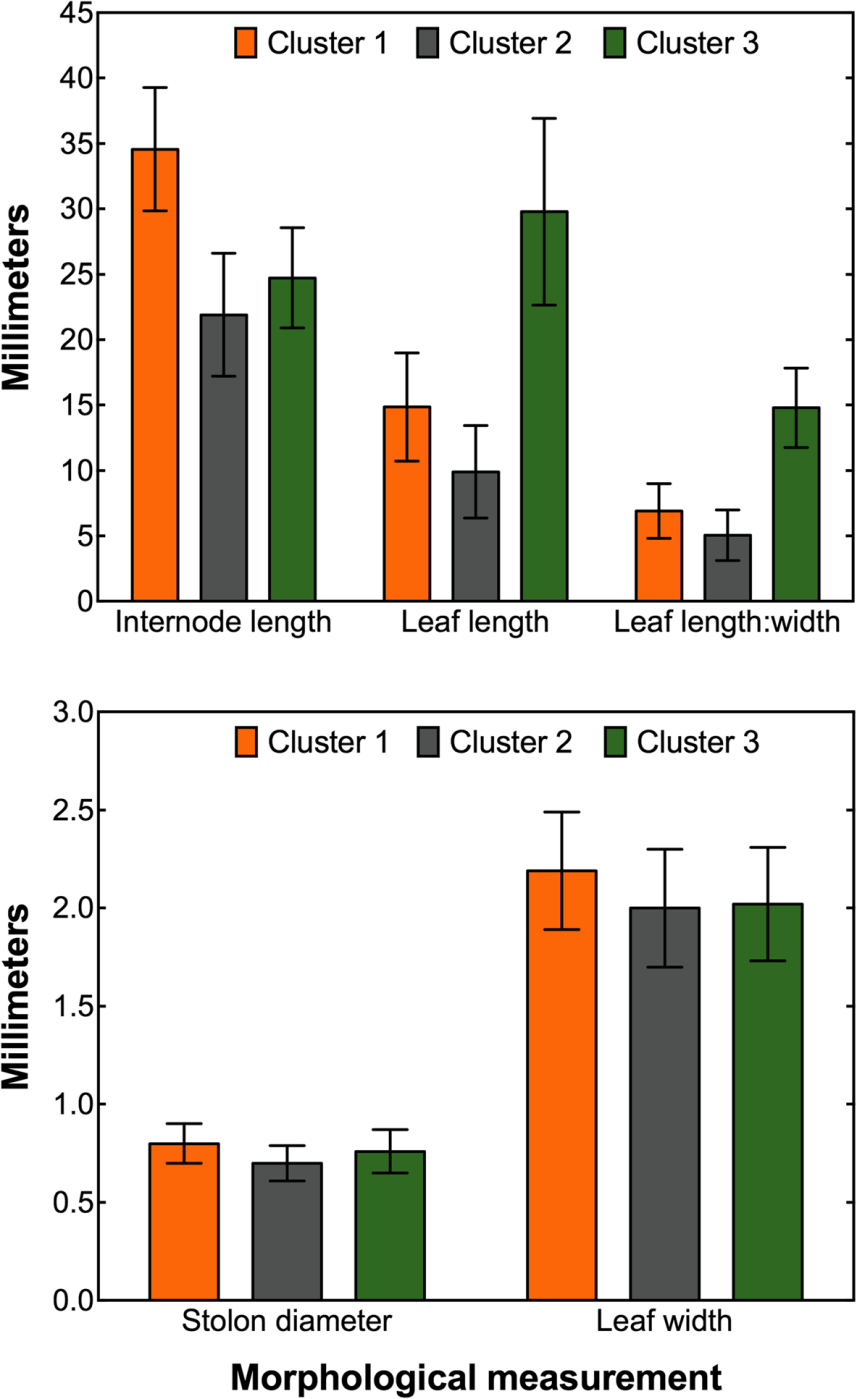
Cluster means and standard deviations for internode length, leaf length, leaf length:width ratio, stolon diameter, and leaf width measurements. Morphological parameters were assessed using methods similar to Roche and Loch [37]. Measurements were made on 52 off-type and desirable hybrid bermudagrass (*Cynodon dactylon* (L.) Pers. x *C. transvaalensis* Burtt-Davy) samples harvested from golf course putting greens in the southeastern United States. Cluster means and standard deviations were generated from the K-means algorithm in SAS Enterprise 6.1 and graphed using Prism 6.0 for Mac. Statistical differences were determined using standard deviations

**Fig. 5:**
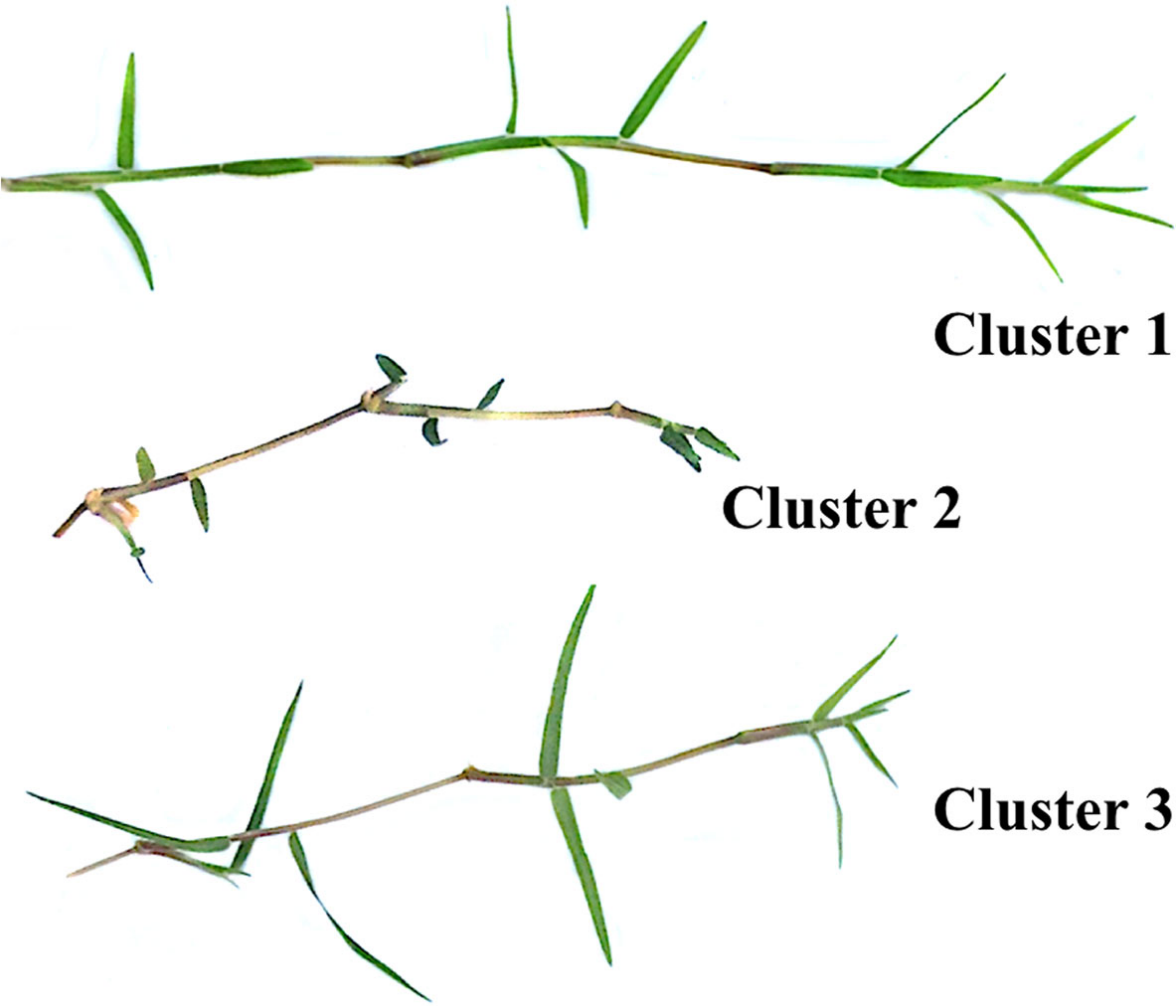
Photographs of bermudagrass samples representative of each morphological cluster. Cluster analysis was performed using a K-means algorithm in SAS Enterprise Guide (Version 6.1, SAS Institute, Cary, NC, USA) with cluster means and standard deviations graphed in Prism (Prism 6 for Mac OS X; GraphPad Software, Inc.) to determine statistical differences. Grasses in cluster one had significantly longer internode lengths than those within clusters two and three. Grasses in cluster three had significantly longer leaves than those in clusters one and two. Figure was generated using Keynote (v6.6.2)

The K-means cluster algorithm yielded three clusters containing 14, 26, and 12 samples, respectively. Cluster one contained nine off-type and five desirable samples, cluster two had 12 off-types and 14 desirables, and cluster three had eight off-types and four desirable samples. The cluster analysis overall expected *R* ^*2*^ was 0.61 with a cubic clustering criterion of −19.36. Cluster means and standard deviations for each morphological assessment are presented in Fig. 4. Internode length, leaf length, and LWR were the only statistically different morphological parameters among clusters (Fig. 4). A representative hybrid bermudagrass sample from each cluster is illustrated in Fig. 5.

The average internode length for grasses in cluster one (34.6 mm) was significantly longer than the grasses within clusters two (21.9 mm) and three (24.7 mm) (Fig. 4). Grasses in cluster three had significantly longer leaves than those grouped in clusters one and two (Fig. 4). The leaf length mean for cluster three was 29.8 mm, compared to 14.9 and 9.9 mm for clusters one and two, respectively (Fig. 4). This relationship was also present in LWR among clusters (Fig. 3). Stolon diameter ranged from 0.7 to 0.8 mm and leaf width ranged from 2.0 to 2.2 mm, with no statistical differences present among clusters for either parameter.

## Discussion

### Genotyping-by-sequencing

Caetano-Anollés [9] and Caetano-Anollés et al. [10] revealed that eight of 16 off-types were genetically divergent from standard cultivars using DAF, leading researchers to conclude that off-types that were not genetically distinct, but were the result of somatic mutations within ‘Tifgreen’ and ‘Tifdwarf’. The inability of GBS, as well as other molecular marker techniques, to distinguish off-types from hybrid bermudagrass cultivars used on putting greens could be the result of aneuploidy within the ‘Tifgreen’-cultivar family (B.M. Schwartz, unpublished data, 2016; [36]). Reasor et al. [36] suggested that aneuploidy could have resulted during the original hybridization of ‘Tifgreen’ or through intense putting green management techniques. It is expected that some variant locations are not going to be sampled by random chance due to the sparse nature of the GBS analysis. This is a limitation of GBS because it cannot determine presence/absence or copy number variations for individual locations that are needed to determine aneuploidy [13]. There were a total of 93,188 variants shared between at least two genotypes (Table 2); however, our experiment only included six biological replications of each standard cultivar from a single geographic location (Tifton, GA). Additional research and replication of this study with more samples will be needed to ascertain which variants can be used to identify standard hybrid bermudagrass cultivars, specifically hybrid ultradwarf cultivars, from one another.

Despite also being a triploid hybrid, ‘Tifway’ bermudagrass has been genetically distinguished from ‘Tifgreen’-cultivar family using SSRs [20, 24, 40] and AFLPs [12, 41]. Arumuganathan et al. [1] reported that ‘Tifway’ had less nuclear DNA content (1.37 ± 0.01 pg/2C) than ‘Tifgreen’ (1.61 ± 0.00 pg/2C) despite having the same number of chromosomes. Furthermore, Reasor et al. [36] hypothesized that this difference in DNA content could also be a result of aneuploidy in the ‘Tifgreen’-cultivar family of hybrid bermudagrass, which includes the hybrid ultradwarf cultivars. This difference in DNA content could also aid in genetic identification between hybrid bermudagrasses using GBS. In addition to the identification of ‘Tifway’ from other triploid hybrids, the ability of GBS to distinguish diploid, triploid, and tetraploid bermudagrasses align with previous efforts to genetically identify these grasses from one another using AFLPs [12, 41] and SSRs [20, 24, 40].

It is not clear why the majority of grasses included in our experiment exhibited variable morphological characteristics while being similar in genotype. The majority of bermudagrass cultivars established on golf course putting greens were selected from other bermudagrass cultivars [36]. The off-type grasses studied in this experiment were also selected from existing cultivars. Differential gene expression driven by epigenetic mechanisms such as DNA methylation, histone modification, and small RNA expression may also be a possible explanation for the genetic similarities among hybrid bermudagrass samples varying in phenotype [39]. Multiple genes control important turfgrass traits and gene expression can be greatly influenced by environment or management practices [14]. Golf course putting greens are intensely managed surfaces subjected to daily mowing (often at heights of cut ≤3 mm), annual aerification and cultivation, as well as treatment with plant growth regulators and silica sand topdressing on a weekly basis. Any of these practices or other environmental influences could cause lasting epigenetic effects that result in the up or down regulation of genes associated with hybrid bermudagrass phenotypic characteristics; however, no research has been conducted on this possibility. Studying changes in gene expression as a result of these maintenance practices could benefit researchers and industry practitioners to better understand how putting green management could potentially lead to the occurrence of phenotypically different off-type grasses in hybrid bermudagrass putting surfaces and generate new hypotheses into how these changes are induced.

### Phenotypic evaluation

The internode length of grasses in this experiment align with Magni et al. [31] who reported an internode length range of 15 to 34 mm on ultradwarf hybrid bermudagrass cultivars used on putting greens. However, Roche and Loch [37] reported internode lengths of 9.4 to 12.5 mm for hybrid bermudagrasses used on putting greens. In our experiment, mean internode length for each cluster was in the uppermost half of the internode length range reported by Magni et al. [31] and greater than the range measured by Roche and Loch [37]. Internode lengths measured in this experiment varied greatly among desirable and off-type grasses as well as grasses measured in other experiments. This is an indication of the amount of phenotypic variability that can occur in individual putting greens as well as from golf course-to-golf course and cultivar-to-cultivar. Differences in internode length within the same putting surface can lead to decreased turfgrass density and reductions in putting surface quality and playability [36]. Similar to the internode length data, leaf length values (and subsequently LWR values) documented in our experiment were far greater than those reported by Roche and Loch [37]. Stolon diameter and leaf width values were similar to those reported by Roche and Loch [37], but less than those reported by Magni et al. [31].

## Conclusions

Off-type grasses reported to have phenotypic differences from standard hybrid bermudagrass cultivars were sampled from golf course putting greens and subjected to GBS and morphological characterization under controlled growth conditions. Genotyping-by-sequencing only distinguished five off-type grasses from standard hybrid bermudagrass cultivars. In addition, GBS failed to completely distinguish standard hybrid bermudagrass cultivars from one another, including ‘Champion’, ‘MiniVerde’, ‘Tifdwarf’, ‘TifEagle’, and ‘Tifgreen’. These results are not unexpected given their common origin. The final bioinformatics analysis did yield 93,188 variants that offer the potential to be useful in distinguishing standard cultivars from one another; however, additional research beyond the scope if this project would be needed to determine which ones are diagnostic. GBS was successful in determining triploid hybrid bermudagrass cultivars from two diploid and tetraploid progenitor samples. Additionally, GBS was also successful in determining triploid hybrid bermudagrass cultivars with lineage to ‘Tifgreen’ from those not developed from ‘Tifgreen’ (e.g., ‘Tifway’). Morphological characteristics varied among sampled grasses that allowed them to be clustered into three distinct phenotypic groups varying predominately in internode and leaf length.
